# Supplementary material for: Preparation and Characterization of Low-Cost Ceramic Membrane Coated with Chitosan: Application to the Ultrafine Filtration of Cr(VI)
Source: Membranes (Basel). 2022 Aug 26;12(9):835. doi: 10.3390/membranes12090835 (PMC9504684; doi:10.3390/membranes12090835)
Supplement: Supplementary file 1 [file membranes-12-00835-s001.zip › membranes-1874095-supplementary.pdf]

Supplementary Information

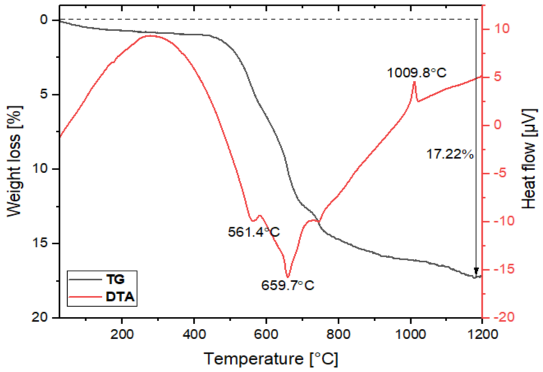

(A)

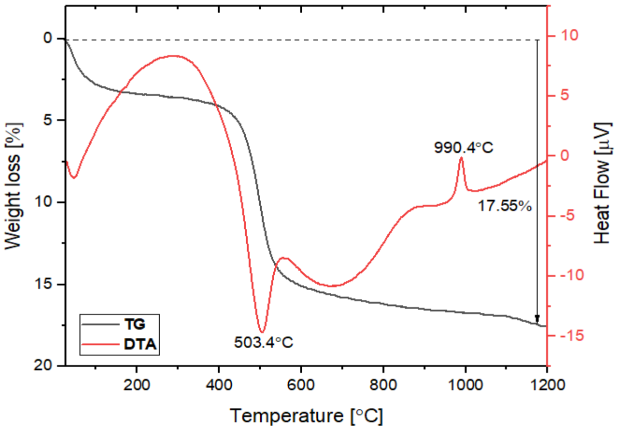

(B)

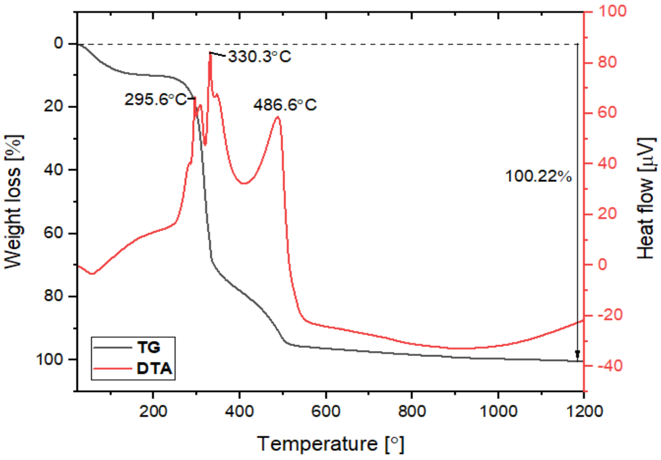

(C)

Figure S1. TG-DTA curves for kaolin (A), clay (B) and starch(C).

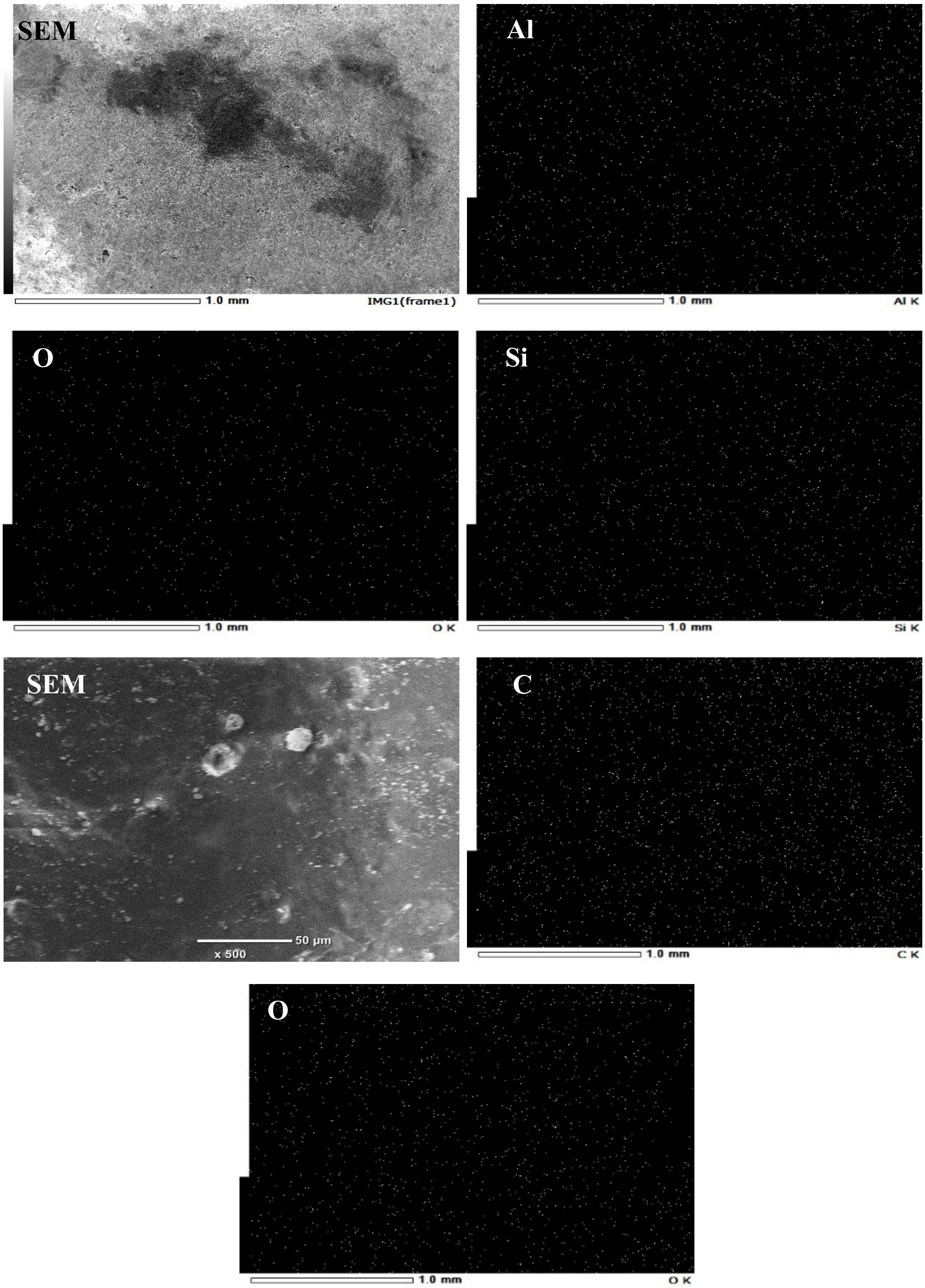

Figure S2. SEM images and mapping images of CMs before coating and after coated with cross-linked chitosan.

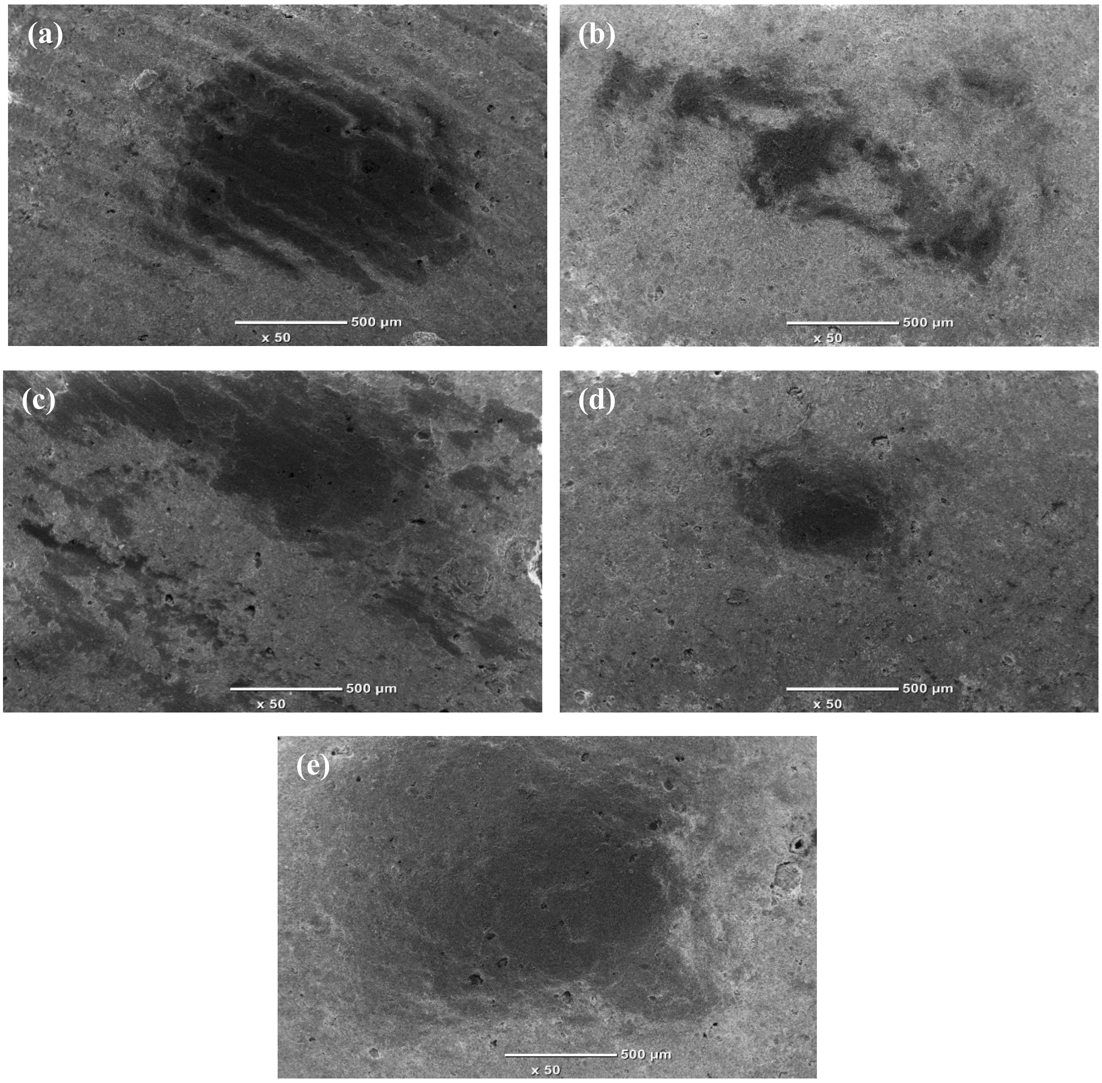

Figure S3. SEM images of CMs surface after sintered at 950℃ (a), 1000℃(b), 1050℃(c), 1100℃(d) and 1150℃(e) for 3h.
